# Understanding barriers to optimal medication management for those requiring long-term dialysis: rationale and design for an observational study, and a quantitative description of study variables and data

**DOI:** 10.1186/s12882-015-0097-2

**Published:** 2015-07-11

**Authors:** Trudi Aspden, Martin J Wolley, Tian M Ma, Edwin Rajah, Samantha Curd, Dharni Kumar, Sophia Lee, Krenare Pireva, Olita Taule’alo, Porsche Tiavale, Angela L Kam, Jun S Suh, Julia Kennedy, Mark R Marshall

**Affiliations:** 1 School of Pharmacy, Faculty of Medical and Health Sciences, The University of Auckland, 85 Park Rd, Auckland, 1142 New Zealand; 2 School of Medicine, University of Queensland, 288 Herston Road, Brisbane, 4006 Australia; 3 Department of Renal Medicine, Counties Manukau District Health Board, Hospital Road, Otahuhu, Auckland, 1640 New Zealand; 4 Marketing Department, Faculty of Business, Auckland University of Technology, 46 Wakefield St, Auckland, 1010 New Zealand; 5 Formerly of the School of Pharmacy, Faculty of Medical and Health Sciences, The University of Auckland, 85 Park Rd, Auckland, 1142 New Zealand; 6 Pharmacy Services, Counties Manukau District Health Board, Hospital Road, Otahuhu, Auckland, 1640 New Zealand; 7 School of Medicine, Faculty of Medical and Health Sciences, The University of Auckland, 85 Park Rd, Auckland, 1142 New Zealand

**Keywords:** Medication adherence, Dialysis, Health literacy, Medication knowledge, Beliefs about medications, Illness perception

## Abstract

**Background:**

Rates of medication non-adherence in dialysis patients are high, and improving adherence is likely to improve outcomes. Few data are available regarding factors associated with medication adherence in dialysis patients, and these data are needed to inform effective intervention strategies.

**Methods/design:**

This is an observational cross-sectional study of a multi-ethnic dialysis cohort from New Zealand, with the main data collection tool being an interviewer-assisted survey. A total of 100 participants were randomly sampled from a single centre, with selection stratified by ethnicity and dialysis modality (facility versus home). The main outcome measure is self-reported medication adherence using the Morisky 8-Item Medication Adherence Scale (MMAS-8). Study data include demographic, clinical, social and psychometric characteristics, the latter being constructs of health literacy, medication knowledge, beliefs about medications, and illness perceptions. Psychometric constructs were assessed through the following survey instruments; health literacy screening questions, the Medication Knowledge Evaluation Tool (Okuyan et al.), the Beliefs about Medication Questionnaire (Horne et al.), the Brief Illness Perception Questionnaire (Broadbent et al.). Using the study data, reliability analysis for internal consistency is satisfactory for the scales evaluating health literacy, medication knowledge, and beliefs about medications, with Chronbach’s α > 0.7 for all. Reliability analysis indicated poor internal consistency for scales relating to illness perceptions. MMAS-8 and all psychometric scores are normally distributed in the study data.

**Discussion:**

This study will provide important information on the factors involved in medication non-adherence in New Zealand dialysis patients. The resulting knowledge will inform long-term initiatives to reduce medication non-adherence in dialysis patients, and help ensure that they are addressing appropriate and evidence based targets for intervention.

**Electronic supplementary material:**

The online version of this article (doi:10.1186/s12882-015-0097-2) contains supplementary material, which is available to authorized users.

## Background

Adherence of patients to prescribed medications is an issue of concern to payers, policymakers, providers and healthcare professionals. Non-adherence is highly prevalent [1], expensive to health systems [2], and associated with poorer outcomes for many chronic diseases [3–5].

In patients with end stage kidney disease, dialysis is an effective treatment for the removal of most uremic toxins, although the majority of patients require a large number of additional medications to control hyperphosphatemia, hypertension, anaemia and other biochemical consequences of their disease. Furthermore, patients often have other comorbid conditions that require treatment. The total drug burden in dialysis patients can be considerable, with the median number of prescribed tablets per day ranging from 12 to 19 in recent observational studies, the highest burden of any chronic disease group [6, 7]. Medication non-adherence within this group is unsurprisingly high (Table 1). A recent systematic review of the literature appraised 19 widely divergent studies, and reported the prevalence of non-adherence to be between 3 to 80 % (depending on definition), with a median of 50 % [8]. In a previous pilot study into medication non-adherence among haemodialysis patients at our institution, non-adherence rates were 33 % and several fold higher amongst New Zealand (NZ) Māori, Pacific Peoples and the elderly [9].

**Table 1:** Recent studies of drug non-adherence rates in dialysis populations

First author of study (year)	Drug/treatment studied	Patient group	Rate of non adherence	Significant correlations
Martins (2013)	Phosphate binders	Haemodialysis (502 patients)	65.7 %	Cerebrovascular disease, higher PTH, comprehension, side effects
Neri (2011)	Oral medications	Haemodialysis (1,238 patients)	48 %	Perceived burden of treatment, number of tablets
Schmid (2009) Systematic review	Oral medications	Haemodialysis (19 studies included)	67 % (range 3–80)	
Karamanidou (2008) Systematic review	Phosphate binders	Dialysis (34 studies included)	Mean 51 % (range 22-74 %)	Younger age, psychosocial beliefs

The consequences of medication non-adherence have not been well established in dialysis populations. Most investigators infer changes in relative mortality risks from suboptimal blood pressure (non-adherence to antihypertensives), suboptimal serum phosphate (non-adherence to phosphate binders), and suboptimal haemoglobin (non-adherence to erythropoietin). Perhaps the most compelling data, however, can be found in a recent study aiming to reduce medication-related problems in haemodialysis patients through an integrated pharmacy program in a large United States dialysis organization. In a retrospective analysis, the mortality risk with an integrated pharmacy program (versus none) was 0.79 (95 % CI, 0.74-0.84), relative rate of hospital admission 0.93 (95 % CI, 0.90-0.96), and relative number of hospital days 0.86 (95 % CI, 0.82-0.90) [10]. There is therefore a strong signal that improved medication adherence might result in clinical benefits for the patient, and lower healthcare resource utilization for the country.

In other patient groups interventional strategies to improve medication adherence have been validated [11], but information specific to dialysis patients is lacking [12]. A major barrier to designing strategies to improve medication adherence in dialysis patients is the lack of knowledge about factors associated with adherence to drugs within this group. Demographic factors, clinical factors and psychosocial factors may all influence medication adherence, but whether these are important in dialysis patients is unclear [13]. There is therefore an exigent need to clarify the drivers of poor medication adherence in this patient group, to inform interventions and optimize their chances of being effective.

In this article, we describe the protocol for an observational study of medication adherence in a large multi-ethnic New Zealand cohort, in which we are studying self-reported medication adherence in relation to a variety of demographic, clinical, social, and psychometric factors. Of note, the psychometric constructs used for this study - and the instruments with which they are assessed – are not well-described in dialysis populations, and distribution of construct scores and the reliability of the instruments cannot be assumed. As part of this article, therefore, we perform and report an interim analysis of the completed study dataset, with a view to ensuring appropriate use of these data in our subsequent statistical modelling.

## Methods/design

### Study aim and hypothesis

The broad aim of this study is to improve our understanding of the factors involved in medication non-adherence in dialysis patients, to inform the development of an intervention strategy. The specific objectives are to estimate self-reported medication adherence in this population, and explore the relationship between this outcome and patients’ medication knowledge and beliefs, health literacy, and illness perceptions. As part of the study, we will explore differences in these relationships by age, ethnicity, dialysis modality (facility based dependent versus home-based independent), and dialysis vintage (duration on dialysis since dialysis inception).

### Study design

This is a cross-sectional study of a sample of prevalent dialysis patients from a single centre.

Demographic and clinical study data were collected from patients’ clinical records (see case report form in Additional file 1), and directly from participant records in the Australia and New Zealand Dialysis and Transplant Registry (www.anzdata.org.au). Patient perceptions and beliefs were assessed using survey instruments administered as an in-person, interviewer-assisted questionnaire (see Additional file 2). As the interviews were not audio-recorded, they were conducted with pairs of interviewers for quality control purposes. Where possible, interviews were conducted in English, but professional interpreters were used if needed in the hospital setting.

The medication discussed in relation to the study was either a self administered erythropoiesis-stimulating agent, an angiotensin converting enzyme inhibitor, or a phosphate binder, depending on each participant’s medication regimen.

Data collection was undertaken from 19^th^ July 2013 to 13^th^ June 2014, with database lock on 9^th^ November 2014.

### Setting

The study setting is the Counties Manukau District Health Board (CMDHB) in Auckland, New Zealand. This is the largest dialysis programme in New Zealand, and provided care at the time of the study to approximately 550 patients, or 22 % of that country’s entire dialysis population [14]. The dialysis population reflects the general population, which is multi-ethnic with a high proportion of NZ Māori and Pacific Peoples, and socioeconomically disadvantaged (www.cmdhb.govt.nz). The prevalence of home dialysis is high in the programme, and 31 % of dialysis patients are on peritoneal dialysis (PD) and 18 % are on home haemodialysis (HD).

### Target population and eligibility criteria

Eligible participants were those with end stage renal disease undergoing dialysis at CMDHB aged ≥16 years old. Exclusion criteria include patients posing logistic or safety risks to interviewers; those suffering acute severe medical illness; those with severe communication difficulties (dysphasia, severe hearing impairment etc.); those who were unable to give direct informed consent.

### Recruitment of participants

Participant selection was by computer-generated random selection from the service census. Selection was stratified by two factors to generate equally sized groups within 6 classifications, as defined by the following strata: recorded ethnicity from clinical records (NZ Māori versus Pacific Peoples versus “other” ethnicity), and location of dialysis (in a facility [in-centre HD] versus at home [home HD or PD]).

### Power calculations

A formal sample size was not calculated for this project, given the exploratory nature of the study. However, the rule of thumb to determine ratio of cases (N) to instrumental variables (m) is N > 50 + 8(m), subject to other factors such as alpha level and expected effect sizes [15, 16]. Given the planned statistical approach, it was anticipated that a sample size of 100 participants in total would provide adequate power for most aspects of analysis.

### Research outcomes and endpoints

The main outcome is self-reported medication adherence, as measured by the Morisky 8-Item Medication Adherence Scale (MMAS-8) [17]. This scale is a reliable and validated instrument, and is one of the most widely used tools to assess patient adherence [18–20]. It comprises eight items that address medication taking behaviour and (intentional and unintentional) adherence. The first seven items in the scale have dichotomous responses (yes/no), and the eighth item has 5 point Likert scale response, from 1 = never to 5 = all the time. The MMAS-8 score ranges from 0 to 8. Those who score less than 6 are considered to have low adherence, and those who score 6 or 7 are considered to have medium adherence, and those who score 8 high adherence.

### Quantitative variables

The following demographic and clinical data were collected:

1. Demographics: We collected data about age, gender, ethnicity (prioritised according to accepted ethnicity data protocols [21–23]), relationship status, home ownership, and household composition, based on questions from the 2006 Statistics New Zealand Census. We assessed the socioeconomic status of the patient cohort using the NZDep score, which combines nine variables from the census that reflect eight domains of deprivation (income, home ownership, social support, employment, academic qualifications, living space, access to a telephone, access to a car). The index provides a score for each meshblock in New Zealand, which are defined geographical areas defined by Statistics New Zealand containing a median number of approximately 87 people in 2006. The NZDep score divides New Zealand into deciles, e.g. a value of 10 indicates the meshblock is in the most deprived 10 % of the New Zealand population, and a value of 1 indicates that the meshblock is in the least deprived [24].
2. Clinical characteristics: We collected data about current modality of dialysis, dialysis vintage, history of previous transplantation, current dialysis dose (expressed as single pool Kt/V), age-adjusted Charlson Co-morbidity Index [25, 26], cause of end stage kidney disease, presence of diabetes mellitus, coronary artery disease, cerebrovascular disease, peripheral vascular disease, and lung disease.

The following study data were collected to evaluate the following psychometric constructs - health literacy, medication knowledge, beliefs about medication, and illness perceptions:

1. Health literacy: We assessed health literacy using a combination of three separate, single-item instruments (see Additional file 2, sections F and G). Each of these instruments has been validated against the S-TOFHLA (Short Test of Functional Health Literacy in Adults) and REALM (Rapid Estimate of Adult Literacy in Medicine) in the general population, and to a lesser degree the dialysis population [27, 28]. The combination of three instruments has been used successfully with health literary assessments in a number of studies [29], including a large one of 1796 Veterans Administration patients [30]. We have previously assessed the validity and reliability of combining the three instruments in this study sample, supporting the use of the average of the individual scores as a single health literacy construct [31]. Those who score three or above on the final combined scale are considered to have marginal or poor health literacy.
2. Medication knowledge: We assessed patients’ knowledge of medications using the Medication Knowledge Evaluation Tool (MKET), an instrument based on the work of McPherson [32], which has been subsequently adapted by Okuyan [33, 34] (see Additional file 2, section B). Knowledge is measured with 7 items, covering medications’ names, intended purpose, intended regimen, intended route of administration, possible side effects, and course of action if side effects occur or a dose is missed. The final score is a summation of seven subscales measured on a 0 or 1 ordinal scale. High medication knowledge is defined as a score ≥ 5.
3. Beliefs about medication: We assessed patients’ perceptions and expectations about their medications using the Beliefs about Medication Questionnaire (BMQ) [35–38] (see Additional file 2, section D). The BMQ contains two separate constructs (generically referred to as the BMQ-Specific subscales), namely the Medication Concerns construct and the Medication Necessity construct. The former represents the concerns of patients around the negative effects for taking their medication, and the latter their beliefs around the necessity of taking it to maintain health. These constructs are sometimes presented as a ratio or differential, although we chose to analyse them separately in this study. The Medications Concerns construct is measured with 5 of the BMQ items (e.g., “I sometimes worry about the long-term effects of my medicines”, “My medicines disrupt my life”), and the Specific Necessity construct with another 5 items (e.g., “My health, at present, depends on my medicines”, “Without my medicines I would be very ill”). Items are rated on 5 point subscales, from 1 = strongly disagree to 5 = strongly agree. A combined high score in the Medications Concerns construct theme means that patients are worried about potential adverse effects of their medications. Conversely, a combined high score in Specific Necessity construct means that patients think their medications are important to them. An additional statement of concern (not included in the Specific Concern construct) is included in the BMQ that asks patients whether they believe their medications to be causing unpleasant side effects, also rated on a 5 point subscale.
4. Illness perceptions: We assessed patients’ perceptions about their health problems using the Brief Illness Perception Questionnaire (BIPQ), an instrument based on the work of Weinman [39] and then Moss-Morris [40], later adapted by Broadbent [41] (see Additional file 2, section E). The BIPQ is a 9-item scale, assessing illness perceptions across 4 interrelated dimensions: cognitive illness representations (items 1–5), emotional illness representations (items 6–8), illness comprehensibility (item 7) and causal representation (item 9). Items are rated on an 11-point Likert-like scale. The BIPQ can be scored in multiple ways, as individual items, as a score within each of the 4 dimensions, or as a total score [41–43]. In the case of scoring, subscales relating to items 3, 4, and 7 should be reversed (Personal communication E Broadbent 15 Jan 2015).
5. Additional qualitative questions were incorporated to supplement some of the study aims (see Additional file 2, Section H). These questions were developed after a literature review, brainstorming by the research team, and discussions with nephrology healthcare professionals within the CMDHB programme.

### Reliability of study instruments

We assessed the reliability of the psychometric instruments by their internal consistency. Internal consistency was defined by the Cronbach’s α statistic, using a score of 0.7 or larger as indicative of a strong level of internal consistency amongst items in the scale, and a score of <0.5 as being indicative of poor internal consistency [44]. Results of the reliability analysis are reported in Table 2. The Cronbach’s α for item subscales within medication adherence, BMQ-specific medication necessity, BMQ-specific medication concerns, and health literacy were 0.77, 0.71, 0.78, and 0.78 respectively. This is indicative of strong level of internal consistency amongst subscales, and supports the retention of all items within each construct. The Cronbach’s α for the subscales within medication knowledge, however, was 0.44 due to the outlying item B7 (See Additional file 2, Section B). By dropping this item from the construct, the Cronbach’s α improved to 0.63, which is closer but nonetheless slightly short of the suggested threshold of 0.7. Despite this marginal internal consistency, a considered judgement was adopted to retain all times in the construct other than for B7. Additionally, Nunally provides support a threshold for Cronbach’s α above 0.6 as being acceptable in the case of an exploratory study [45].

**Table 2:** Internal consistency of construct item subscales

Construct	Instrument item	Subscale mean if item deleted	Subscale variance if item deleted	Corrected item-total correlation	Squared multiple correlation	Cronbach’s α if item deleted
*Medical Adherence* Cronbach’s α = 0.77	C1	5.08	3.43	0.57	0.40	0.72
	C2	4.79	3.58	0.49	0.27	0.74
	C3	4.66	3.90	0.39	0.28	0.75
	C4	4.79	3.66	0.44	0.25	0.75
	C5	4.53	4.17	0.40	0.19	0.76
	C6	4.63	3.71	0.56	0.41	0.73
	C7	4.76	3.76	0.40	0.21	0.75
	C8	4.91	3.50	0.51	0.39	0.73
*Health Literacy* Cronbach’s α = 0.78	F1	5.45	7.62	0.62	0.39	0.70
	F2	4.84	7.69	0.60	0.36	0.72
	F3	5.45	8.23	0.64	0.41	0.69
*Medication Knowledge (Original)* Cronbach’s α = 0.44	B1	4.45	0.90	0.27	0.39	0.37
	B2	4.14	0.91	0.26	0.18	0.38
	B3	3.83	1.15	0.51	0.62	0.34
	B4	3.86	1.12	0.36	0.51	0.36
	B5	4.69	1.08	0.33	0.24	0.35
	B6	3.90	1.03	0.42	0.35	0.31
	B7	3.90	1.60	−0.39	0.24	0.63
*Medication Knowledge – (Modified, B7 omitted)* Cronbach’s α = 0.63	B1	3.55	0.97	0.41	0.28	0.57
	B2	3.24	1.05	0.32	0.16	0.62
	B3	2.93	1.35	0.48	0.62	0.58
	B4	2.97	1.32	0.35	0.51	0.59
	B5	3.79	1.24	0.37	0.24	0.58
	B6	3.00	1.21	0.42	0.35	0.57
*Beliefs about Medication - Medical Necessity* Cronbach’s α = 0.71	D1	16.93	5.96	0.42	0.23	0.73
	D3	17.20	4.12	0.61	0.40	0.66
	D4	16.87	5.39	0.47	0.33	0.71
	D7	17.06	5.27	0.58	0.38	0.67
	D10	16.94	6.06	0.51	0.31	0.70
*Beliefs about Medication - Medical Concerns* Cronbach’s α = 0.78	D2	11.27	13.57	0.54	0.33	0.74
	D5	10.67	12.47	0.61	0.40	0.72
	D6	11.05	13.89	0.49	0.27	0.76
	D8	11.46	14.09	0.54	0.30	0.74
	D9	10.91	13.92	0.60	0.36	0.73
*Illness Perception – Cognitive (Original)* Cronbach’s α = 0.41	E1	18.9	37.4	0.31	0.16	0.28
	E2	17.0	46.6	0.10	0.04	0.44
	E3^a^	22.2	32.4	0.38	0.16	0.20
	E4^a^	24.9	52.7	0.03	0.04	0.45
	E5	20.2	37.0	0.22	0.11	0.36
*Illness Perception – Cognitive (Modified, E2 omitted)* Cronbach’s α = 0.44	E1	10.1	29.5	0.30	0.15	0.32
	E3^a^	13.4	24.9	0.37	0.15	0.22
	E4^a^	16.1	43.5	0.02	0.04	0.52
	E5	11.4	26.5	0.29	0.09	0.33
*Illness Perception – Cognitive (Modified, E4 omitted)* Cronbach’s α = 0.45	E1	18.1	32.9	0.37	0.14	0.28
	E2	16.1	43.5	0.09	0.04	0.52
	E3^a^	21.3	30.4	0.35	0.13	0.28
	E5	19.3	33.5	0.23	0.11	0.41
*Illness Perception – Cognitive (Modified, E2 and E4 omitted)* Cronbach’s α = 0.52	E1	9.2	25.4	0.35	0.13	0.38
	E3^a^	12.5	23.2	0.34	0.12	0.41
	E5	10.5	23.3	0.31	0.09	0.46
*Illness Perception – Emotional* Cronbach’s α = 0.52	E6	5.90	10.1	0.35	0.12	n/a
	E8	6.90	12.0	0.35	0.12	n/a

^a^Scale reversed for these items (Personal communication E. Broadbent 15 Jan 2015

**Table 3:** Summary of scores for the final constructs of medication adherence, medication knowledge, health literacy, beliefs about medication, and illness perception, reported as mean (standard deviation)

Construct	Score
Medication adherence	5.5 (2.2)
Medication knowledge	4.3 (1.5)
Health literacy^a^	2.6 (1.3)
Beliefs about medication (BMQ)	
Medication necessity	21.3 (2.8)
Medication concerns	13.8 (4.5)
Illness perception (BIPQ)^b^	
Cognitive dimension	32.2 (5.6)
Emotional dimension	12.8 (5.5)
Illness comprehensibility	2.02 (2.8)

^a^Incomplete data for 1 patient - excluded

^b^Incomplete data for 3 patients - excluded

For BIPQ cognitive dimension, two item subscales (E3 & E4) were reversed for scoring. For illness comprehensibility, one item subscale (E7) was reversed for scoring (Personal communication E. Broadbent 15 Jan 2015)

The internal consistency of the subscales for the BIPQ emotional and cognitive dimensions were poor, although the reliability of subscales within the cognitive dimension improved a little by dropping the outlying items E2 and E4 (See Additional file 2, Section E). Even after these manoeuvres, however, Cronbach’s α did not reach the acceptable threshold of 0.6.

### Distribution of scores for psychometric constructs

We assessed the scores from the MMAS-8 and psychometric constructs for normality, skewness and kurtosis. A strictly quantitative description of the various scores is provided in Table 3, without any accompanying interpretation or insights. Inspection of the z-scores for both the kurtosis criteria suggest the scores are well within the +/−1.96 cut-off points (*p* ≤ 0.05) for normality [46]. The z-scores for skewness are below the +/−1.96 (*p* ≤ 0.05) criteria for the knowledge and concerns scores, while the necessity and total adherence scores for skewness are below the +/−2.58 cut-off (*p* ≤ 0.01). Since a sample of 100 fits within the guideline of a small sample, the skewness scores suggest that the dataset is borderline but acceptable for least squares regression, especially in the context of multivariate data analysis [47].

### Statistical analysis

**Fig. 1:**
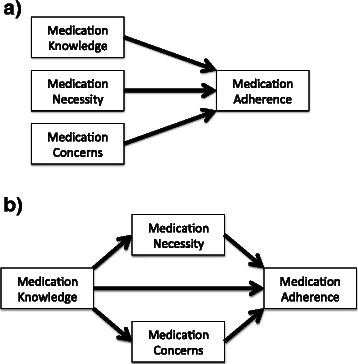
Examples of extended regression analyses planned for these study data. Panel (**a**) exemplifies conventional regression analysis, and panel (**b**) exemplifies mediation regression analysis

Simple comparisons will be made by Chi-square test, Student’s *t*-test or their non-parametric equivalents as appropriate. Exploratory modelling will be performed using multivariate regression analysis, using a number of specific techniques. These will include conventional regression analysis, and also mediation, moderation, and conditional process analysis (see an example in Fig. 1) [48]. Scores for psychometric constructs can be modelled as either continuous variables (where higher scores indicate stronger beliefs or perceptions in the construct represented by the scale), or by dichotomizing at the scale midpoint or some other point of discrimination [37]. While the latter is a convenient way of categorizing respondents, we will attempt to use the continuous scale analyses as this provides richer information that is lost when scales are dichotomized. Statistical significance of associations will be attributed to findings if the two-tailed *P* value is <0.05.

Qualitative data will be analyzed using a general inductive/thematic approach [49].

### Ethical considerations

The study protocol has been reviewed and approved by the National (NZ) Health and Disability Ethics Committee (IORG0000895) and the Counties Manukau Institutional Review Board (FWA00021560), and the University of Auckland Human Participants Ethics Committees (IRB00009352). Informed consent was gained from all participants.

## Discussion

This study will provide important information on the factors involved in medication non-adherence in New Zealand dialysis patients. The resulting knowledge will inform long-term initiatives to reduce medication non-adherence in dialysis patients, and help ensure that they are addressing appropriate and evidence based targets for intervention.

Important strengths of this study include the use of well-validated study instruments, and a study sample that is inclusive of different ethnicities and importantly modalities/locations of dialysis. These will allow us to model links between demographics, environment, and psychometric characteristics when assessing their individual effects on medication adherence. However, there are several limitations of this study. Firstly, the study design is based upon interviewing each patient about one medication that they are taking, a pragmatic compromise that was accepted by the research team given the diversity of different medication regimens in the study sample. It is possible that adherence will differ between different types of medication, which is something that cannot be assessed in this study, and could conceivably be a source of unmeasured confounding. Secondly, the sample size is modest, especially given the diversity of the sample population. Notwithstanding, 100 participants is probably adequate given the novel regression techniques that are planned, where we will model 3 or 4 variables as either instrumental or mediating factors, and the rest as effect modifiers. Finally, the qualitative interviews were not recorded because of feasibility/resource constraints, and also because this part of the study was to supplement study aims that (in the great majority) were being addressed by the survey tool. In an ideal world, verbatim transcription of recorded interviews would be undertaken, as is the standard recommended practice. This compromise can be expected to reduce the strength and number of insights from the qualitative portion of the study.

Patient-related factors are well known to influence medication adherence. Some of these factors are purely unintentional; such as personality, attentional and coping style, comprehension difficulties, access to medications etc. [50, 51]. Other factors are intentional, and some of these relate closely to patient perceptions (Table 4). Patients’ pre-existing beliefs about illness and medical treatment are likely to affect their motivation to adhere to prescribed treatment [40]. Traditionally research has focused on perceptions of the need for treatment and concerns about potential adverse effects (the necessity-concerns framework) [52]. Within this framework, a stronger perception of necessity for treatment is associated with higher adherence across disparate patient groups with chronic diseases. Similarly, fewer concerns about treatment are also associated with greater adherence [52, 53]. These perceptions may be influenced by factors such as ethnicity and cultural beliefs, [54] health literacy and other psychosocial factors [13]. Limited health literacy is particularly widespread in dialysis patients, and associated with reduced access to healthcare [55, 56]. Health literacy is a direct and indirect determinant of medication adherence, and improving literacy may afford clinician better opportunities to communicate with patients about their medication [57, 58]. The impact of environmental factors, for instance the location and modality of dialysis, are not as well understood in regards to the extent to which they influence medication adherence [59, 60]. In general, environmental factors are potentially more amenable to modification than patient factors, and are therefore important to investigate. Despite this, interventions to improve medication adherence often address patient-related barriers, and less often condition, therapy, and socioeconomic factors [61]. For instance, medication reconciliation exercises often stall at initiation, as patients are unable to recall the names of their medications. A potentially inexpensive strategy for this problem may be to simplify medication names to improve pill identification [62].

**Table 4:** Examples of possible reasons for medication non-adherence with potential risk factors and intervention strategies

Example reasons for medication non-adherence		Possible risk factors	Possible intervention strategy
Unintentional	Difficulty with access to medications	Financial restrictions	Social support
		Socioeconomic	Government support
	Poor comprehension	Language barriers	Multi-lingual information availability
		Literacy	Translators
		Low education level	Family assistance
		Visual impairment	Evaluation of available materials and development of new resources
	Forgetting medications	Attention style	Family support
		Poor cognitive function	Physical or electronic reminders
			Blister packed medications
	Poor tolerability	Pill burden	Minimise dosing where possible
		Frequent dosing	Simplify drug regimen
Intentional or related to health beliefs	Low perception of medication importance	Low health literacy	Education
		Age	Health psychology/psychiatry input
		Depression	Culturally appropriate information
		Cultural beliefs	Establishing goals of treatment
	High perception of potential harm from medications	Low health literacy	Education
		Cultural beliefs	Culturally appropriate information

There is limited evidence for strategies to improve adherence to therapy in dialysis patients. In a systematic review of trials to improve general adherence to treatment in haemodialysis patients, Matteson and Russell summarized 8 trials, finding that 6/8 trials found modest improvements in adherence with intervention, and that most successful interventions relied on cognitive or cognitive/behavioural intervention strategies [12]. Overall evidence was however limited by small sample size, homogenous samples, short intervention periods and a high baseline rate of poor adherence.

Current health outcomes for patients on dialysis are significantly poorer than that of the general population, with a median survival after dialysis inception of 4.2 years in New Zealand [63] and a significantly impaired quality of life [64]. Given the high prevalence of medication non-adherence in dialysis patients and the potential for improved outcomes [10], improving our knowledge about factors involved in medication adherence in these patients is of vital importance.

## Additional files

Additional file 1:
**Clinical Data Case Report Form Version 1.1 29.04.2013.**

Additional file 2:
**In-person, interviewer-assisted questionnaire.**

## Abbreviations

BIPQ: Brief Illness Perception Questionnaire
BMQ: Beliefs about Medication Questionnaire
MKET: Medication Knowledge Evaluation Tool
CKD: Chronic kidney disease
CMDHB: Counties Manukau District Health Board
NZ: New Zealand
S-TOFHLA: Short Test of Functional Health Literacy in Adults
REALM: Rapid Estimate of Adult Literacy in Medicine
MMAS-8: Morisky 8-item Medication Adherence Scale
