# Supplementary material for: Understanding barriers to optimal medication management for those requiring long-term dialysis: rationale and design for an observational study, and a quantitative description of study variables and data
Source: BMC Nephrol. 2015 Jul 11;16:102. doi: 10.1186/s12882-015-0097-2 (PMC4499205; doi:10.1186/s12882-015-0097-2)
Supplement: Additional file 1: — Clinical Data Case Report Form Version 1.1 29.04.2013. [file 12882_2015_97_MOESM1_ESM.pdf]

Clinical Data CRF
Version 1.1
29.04.2013

Participant Number | | |

1. Date of dialysis inception

__/__/____

2. Current modality of dialysis (single option)

Facility HD ☐

Home HD ☐

PD ☐

3. Current age-adjusted Charlson Co-morbidity Index[1,2]

____

4. Current NZDep score

____

5. Current medical comorbidity (multiple options)

Diabetes Mellitus ☐

Coronary artery disease ☐

Cerebrovascular disease ☐

Peripheral vascular disease ☐

Lung disease ☐

---

[1] Charlson ME, Pompei P, Ales KL, MacKenzie CR: **A new method of classifying prognostic comorbidity in longitudinal studies: development and validation**. *J Chronic Dis* 1987, **40**(5):373-383.

[2] Hall WH, Ramachandran R, Narayan S, Jani AB, Vijayakumar S: **An electronic application for rapidly calculating Charlson comorbidity score**. *BMC Cancer* 2004, **4**:94
