# Supplementary material for: Understanding barriers to optimal medication management for those requiring long-term dialysis: rationale and design for an observational study, and a quantitative description of study variables and data
Source: BMC Nephrol. 2015 Jul 11;16:102. doi: 10.1186/s12882-015-0097-2 (PMC4499205; doi:10.1186/s12882-015-0097-2)
Supplement: Additional file 2: — In-person, interviewer-assisted questionnaire. [file 12882_2015_97_MOESM2_ESM.pdf]

Participant Number: ☐☐☐

Researcher Copy

SECTION A: *Demographics

1. **Age** ☐☐
2. **Gender**
   ☐ Male ☐ Female
3. **Which ethnic group/s do you belong to? – *Mark all the spaces that apply to you.***
   ☐ NZ European
   ☐ Māori
   ☐ Samoan
   ☐ Cook Island Māori
   ☐ Tongan
   ☐ Niuean
   ☐ Chinese
   ☐ Indian
   ☐ Other - *please state*--
4. **What is your highest secondary school qualification?**
   ☐ None
   ☐ NZ School Certificate in one or more subjects *or*
   National Certificate Level 1 *or*
   NCEA Level 1
   ☐ NZ Sixth Form Certificate in one or more subjects *or*
   National Certificate Level 2 *or*
   NZ UE before 1986 in one or more subjects *or*
   NCEA Level 2
   ☐ NZ Higher School Certificate *or*
   Higher Leaving Certificate *or*
   NZ University Bursary/Scholarship *or*
   National Certificate Level 3 *or*
   NCEA Level 3 *or*
   NZ Scholarship
   ☐ Other secondary schooling qualification gained in NZ – *please state*

   ☐ Other secondary schooling qualification gained overseas
5. **Apart from secondary school qualifications, do you have another completed qualification?**
   ☐ Yes ☐ No
   If yes, please state --

   ----

6. **Living status – *mark as many spaces as you need to show all the people living in the same household as you.***

☐ Husband, wife or partner
☐ Mother and/or father
☐ Son(s) and/or daughter(s)
☐ Brother(s) and/or sister(s)
☐ Flatmate/s
☐ Other, for example *GRANDMOTHER, MOTHER AND/OR FATHER IN LAW – please state*

----

7. **Number of regular medications taken** --

Participant Number: ☐☐☐

SECTION B: *Medication Knowledge Evaluation Tool

1. **Can you list the names of all medications you are currently taking?**

    ☐ Correct if participant states either generic or brand name (1)

    ☐ Participant does not know (0)

----

2. **Can you tell me why you are taking this medication?**

    ☐ Participant correctly states reason for administration of medication (1)

    ☐ Participant does not know (0)

----

3. **Do you know how to take your medicine?**

    ☐ Participant can correctly describe administration method for this medication (e.g. tablet; swallowing the tablet whole with plenty of water) (1)

    ☐ Participant does not know (0)

----

4. **Do you know when to take your medicine?**

    ☐ Correct if participant correctly describes when to take this medication (1)

    ☐ Participant does not know (0)

----

5. **Do you know the possible side effects of your medicine?**

    ☐ Correct if participant can state medication side effects, including those not experienced by patient (1)

    ☐ Participant does not know (0)

----

6. Do you know what to do if your medication's side effects occur?

☐ Correct if participant states they would call their physician/pharmacist, stop taking the medication, or other self-management intervention methods when faced with side effects (1)

☐ Participant does not know (0)

----

7. Do you know what to do if you miss a dose of your medicine?

☐ Participant says he or she never forgets a dose, he or she takes the next scheduled dose, or he or she calls physician or pharmacist (1)

☐ Incorrect if participant does not know or declares he or she doubles up on doses (0)

Participant Number: ☐☐☐

SECTION C: *Morisky Medication Adherence Scale

"Having to take lots of medicines every day can be hard and people usually find ways of using their medications which suit them. We are interested in finding out how you use your medications."

1. **Do you sometimes forget to take your medications?**

Never/rarely   Once in a while   Sometimes   Usually   All the time

2. **People sometimes miss taking their medicines for reasons other than forgetting. Thinking over the past two weeks, were there any days when you did not take your medicine?**

Never/rarely   Once in a while   Sometimes   Usually   All the time

3. **Have you ever cut back or stopped taking your medicine without telling your doctor because you felt worse when you took it?**

Never/rarely   Once in a while   Sometimes   Usually   All the time

4. **When you travel or leave home, do you sometimes forget to bring along your medicines?**

Never/rarely   Once in a while   Sometimes   Usually   All the time

5. **Did you take all of your medicines yesterday?**

☐ Yes   ☐ No

6. **When you feel like your symptoms are under control, do you sometimes stop taking your medicines?**

Never/rarely   Once in a while   Sometimes   Usually   All the time

7. **Taking medicine every day is a real inconvenience for some people. Do you ever feel hassled about sticking to your treatment plan?**

Never/rarely   Once in a while   Sometimes   Usually   All the time

8. **How often do you have difficulty remembering to take all of your medicine?**

Never/rarely   Once in a while   Sometimes   Usually   All the time

Participant Number: | | | |

SECTION D: ***Beliefs about Medicines Questionnaire**

"We would like to ask you about your views about the medicines prescribed to you. I will read statements that other people have made about their medications and we would like you to tell us how much you agree or disagree with them. There are no right or wrong answers. We are just interested in your own views."

1. **My health at present, depends on my medicines**. (N)

Strongly Agree Agree Neither agree nor disagree Disagree Strongly Disagree

2. **Having to take medicines worries me**. (C)

Strongly Agree Agree Neither agree nor disagree Disagree Strongly Disagree

3. **My life would be impossible without my medications.** (N)

Strongly Agree Agree Neither agree nor disagree Disagree Strongly Disagree

4. **Without my medicines I would be very ill/sick**. (N)

Strongly Agree Agree Neither agree nor disagree Disagree Strongly Disagree

5. **I sometimes worry about the long-term effects of my medicines**. (C)

Strongly Agree Agree Neither agree nor disagree Disagree Strongly Disagree

6. **My medicines are a mystery to me.** (C)

Strongly Agree Agree Neither agree nor disagree Disagree Strongly Disagree

7. **My health in the future will depend on my medicines**. (N)

Strongly Agree Agree Neither agree nor disagree Disagree Strongly Disagree

8. **My medicines disrupt my life**. (C)

Strongly Agree Agree Neither agree nor disagree Disagree Strongly Disagree

9. **I sometimes worry about becoming too dependent on my medicines**. (C)

Strongly Agree Agree Neither agree nor disagree Disagree Strongly Disagree

10. **My medicines protect me from becoming worse.** (N)

Strongly Agree Agree Neither agree nor disagree Disagree Strongly Disagree

11. **My medicines give me unpleasant side effects**. (C)

Strongly Agree Agree Neither agree nor disagree Disagree Strongly Disagree

Participant Number: [ ][ ][ ]

SECTION E: *Brief Illness Perception Questionnaire

1. How much does your illness affect your life?

No affect at all 0 1 2 3 4 5 6 7 8 9 10 *Severely affects my life*

2. How long do you think your illness will continue?

A very short time 0 1 2 3 4 5 6 7 8 9 10 *Forever*

3. How much control do you feel you have over your illness?

Absolutely no control 0 1 2 3 4 5 6 7 8 9 10 *Extreme amount of control*

4. How much do you think your treatment can help your illness?

Not at all 0 1 2 3 4 5 6 7 8 9 10 *Extremely helpful*

5. How much do you experience symptoms from your illness?

No symptoms at all 0 1 2 3 4 5 6 7 8 9 10 *Many severe symptoms*

6. How concerned are you about your illness?

Not at all concerned 0 1 2 3 4 5 6 7 8 9 10 *Extremely concerned*

7. How well do you feel you understand your illness?

Don't understand at all 0 1 2 3 4 5 6 7 8 9 10 *Understand very clearly*

8. How much does your illness affect you emotionally? (E.g. does it make you angry, scared, upset, depressed?)

Not affected emotionally 0 1 2 3 4 5 6 7 8 9 10 *Extremely affected emotionally*

9. Please list in ranked-order, the three most important factors that you believe caused your illness.

The most important cause for me is:

1) --

2) --

3) --

Participant Number: | | | |

SECTION F: ***Single Item Literacy Screener**

1. **How confident are you at filling out medical forms by yourself?**

Always Quite a bit Somewhat A little bit Not at all

SECTION G: ***Additional Screeners**

1. **How often do you have someone (like a family member, friend, hospital/clinic worker or caregiver) help you read hospital materials?**

Always Often Sometimes Occasionally Never

2. **How often do you have problems learning about your medical condition because of difficulty understanding written information?**

Always Often Sometimes Occasionally Never

Participant Number: ☐☐☐

SECTION H: ***Additional Questions**

1. **What are your expectations of the health care services provided to you?** ☐ P ☐ N

   "For example: when you go to a tyre garage, you expect them to fix your puncture and check your wheel alignment."

----

2. **Who manages your medications?** ☐ P ☐ N

   "For example: a mother looks after her 2 year old son's medications."

----

3. **Thinking about your experience with kidney disease, what do you believe made it hard for you to manage your condition?** ☐ P ☐ N

   "For example: growing your tomatoes can be hard because the slugs love to eat them."

----

4. **Thinking about your kidney disease, is there anything that you feel you would like to learn more about?** ☐ P ☐ N

   - ☐ Medications
   - ☐ Medical condition/s
   - ☐ How to look after yourself to stay healthy

Participant Number: ☐☐☐

5. How would you like to learn about the things you just talked about in question 4? ☐ P ☐ N

☐ Written information, such as pamphlets
☐ A video or dvd
☐ A website with both written information and videos
☐ A face to face session with a pharmacist

Thank you for your time and help
